# A vascular smooth muscle-specific integrin-α8 Cre mouse for lymphatic contraction studies that allows male-female comparisons and avoids visceral myopathy

**DOI:** 10.3389/fphys.2022.1060146

**Published:** 2023-01-12

**Authors:** Michael J. Davis, Hae Jin Kim, Min Li, Scott D. Zawieja

**Affiliations:** Department of Medical Pharmacology and Physiology, University of Missouri School of Medicine, Columbia, MO, United States

**Keywords:** lymphatic smooth muscle, *Myh11-CreER^T2^*, *Itga8-CreER^T2^*, integrin alpha-8, L-type voltage-gated calcium channel, *CaV1.2*, contraction, *Rosa26mTmG*

## Abstract

**Introduction:** The widely-used, tamoxifen-inducible, smooth muscle (SM)-specific Cre, *Myh11-CreER^T2^*, suffers from two disadvantages: 1) it is carried on the Y-chromosome and thus only effective for gene deletion in male mice, and 2) it recombines in both vascular and non-vascular SM, potentially leading to unwanted or confounding gastrointestinal phenotypes. Here, we tested the effectiveness of a new, SM-specific Cre, based on the integrin α8 promoter (*Itga8-CreER^T2^*), that has been recently developed and characterized, to assess the effects of *Cav1.2* deletion on mouse lymphatic SM function.

**Methods:** *Cav1.2* (the L-type voltage-gated calcium channel) is essential for lymphatic pacemaking and contraction and its deletion using either *Myh11-CreER^T2^* or *Itga8-CreER^T2^* abolished spontaneous lymphatic contractions. Mouse lymphatic contractile function was assessed using two *ex vivo* methods.

**Results:** *Myh11-CreER^T2^*; *Cav1.2* ^*f/f*^ mice died of gastrointestinal obstruction within 20 days of the first tamoxifen injection, preceded by several days of progressively poor health, with symptoms including weight loss, poor grooming, hunched posture, and reduced overall activity. In contrast, *Itga8-CreER^T2^*; *Cav1.2* ^*f/f*^ mice survived for >80 days after induction and were in normal health until the time of sacrifice for experimental studies. *Cav1.2* deletion was equally effective in male and female mice.

**Discussion:** Our results demonstrate that *Itga8-CreER* ^*T2*^ can be used to effectively delete genes in lymphatic smooth muscle while avoiding potentially lethal visceral myopathy and allowing comparative studies of lymphatic contractile function in both male and female mice.

## Introduction

The spontaneous contractions of collecting lymphatic vessels are the basis of active lymphatic pumping (Davis et al., 2012; Scallan et al., 2016) and responsible for a substantial component of lymphatic transport (Olszewski and Engeset, 1980). These contractions are initiated by action potentials in lymphatic smooth muscle, triggering the opening of L-type voltage-gated calcium channels (VGCCs), which allow calcium entry for the activation of myosin light chain kinase (Wang et al., 2009; Nepiyushchikh et al., 2011), subsequent MLC_20_ phosphorylation, and actomyosin shortening.

The well-documented inhibition of spontaneous lymphatic contractions by dihydropyridine antagonists of L-type VGCCs (Hollywood et al., 1997; van Helden, 1993; Imtiaz et al., 2007; von der Weid et al., 2008; von der Weid and Zawieja, 2004; Telinius et al., 2014; Cotton et al., 1997) provides support for the contention that these channels are critical for spontaneous lymphatic contractions. In a recent study, our laboratory demonstrated that smooth muscle (SM)-specific knock out of *Cacna1c* (hereafter referred to as *Cav1.2*), the gene encoding the SM variant of the L-type VGCC (Ertel et al., 2000), completely eliminated spontaneous contractions in mouse popliteal and inguinal-axillary lymphatic collectors (To et al., 2020). Residual contractile activity in those vessels consisted only of small amplitude (1–4 μm), unsynchronized contractions that were likely insufficient to generate propulsive lymphatic flow. That study utilized a tamoxifen-inducible, SM-specific Cre, *Myh11-CreER^T2^*, developed by Offermanns and colleagues (Wirth et al., 2008), which is now used widely for vascular and lymphatic studies (Retailleau et al., 2015; Castorena-Gonzalez et al., 2018a; Chennupati et al., 2019; Zawieja et al., 2019). Two disadvantages of *Myh11-CreER^T2^* are that: 1) it is carried on the Y-chromosome and thus only effective for gene deletion in male mice, and 2) it recombines in both vascular and non-vascular SM. Unintended deletion of *Cav1.2* from gastrointestinal SM with *Myh11-CreER^T2^* led to the death of most mice from intestinal obstruction/impaction by day ∼20 after the first tamoxifen injection (To et al., 2020). Death was preceded by several days of progressively poor health, with symptoms that included weight loss, poor grooming, hunched posture, and reduced overall activity of the animals. For this reason, we typically sacrificed the mice between days 15 and 17 to harvest lymphatic vessels for *ex vivo* assessment of contractile function (To et al., 2020). However, the uncertain health status of the mice at that time point was an unavoidable and potentially confounding contributor to the observed changes in lymphatic contractile function given the well-established inhibition of contractility in disease states (Von der Weid and Rehal, 2010; Liao et al., 2011; Zawieja et al., 2012; Cromer et al., 2015). Gene deletion from gastrointestinal SM is a disadvantage shared by the two other commonly used inducible SM-specific Cre lines, *Sm22-CreER^T2^* and *Acta2-CreER^T2^* (Kuhbandner et al., 2000; Grcevic et al., 2012). A similar degree of unintended mortality was previously reported by Moosmang et al. (Moosmang et al., 2003), who used *Sm22-CreER^T2^* (Kuhbandner et al., 2000) to delete *Cav1.2* from arterial smooth muscle in order to study the consequences for systemic vascular function. In their study, they observed that the *Cav1.2*-deficient mice “show [ed] general signs of severe illness (lowered activity, relieving posture)” between 21 and 28 days after the first tamoxifen injection, and died between 28 and 35 days after induction, probably due to “complete ileus (bowel paralysis) combined with urinary retention” and subsequent “complications of the ileus (peritonitis, shock)” (Moosmang et al., 2003).

Here, we tested the effectiveness of a new vascular SM-specific Cre, based on the integrin α8 promoter (*Itga8-CreER^T2^*), recently developed and characterized by Miano and colleagues (Warthi et al., 2022), to assess the effects of *Cav1.2* deletion on lymphatic function. This Cre was demonstrated to be much more specific for vascular than visceral SM based on multiple indices, including the important advantage that deletion of genes such as *Srf* (*Itga8-CreER^T2^*;*Srf* ^*f/f*^ mice) avoided an otherwise lethal gastrointestinal phenotype (Warthi et al., 2022). *Itga8-CreER^T2^* can be used to delete genes in both male and female mice, potentially allowing studies of sex differences. Recombination in lymphatic smooth muscle was shown to be somewhat less efficient than with the *Myh11*-based Cre, based on GFP expression, as lymphatic vessels from *Itga8-CreER^T2^* *;Rosa26mTmG* ^*f/f*^ mice expressed GFP in 65% of lymphatic SM cells compared to 90% of lymphatic SM cells in *Myh11-CreER^T2^* *;Rosa26mTmG* ^*f/f*^ mice [Figure 3 in (Warthi et al., 2022)]. However, recombination efficiencies based on the excision of stop floxed cassettes in the *mTmG* reporter and an inducible *Myocardin* transgene as well as a floxed *Srf* mouse (Miano et al., 2004) were more comparable to those when *Myh11-CreER^T2^* was used (Warthi et al., 2022). The goal of the present study was to use *Itga8-CreER^T2^* to delete *Cav1.2* and assess the impact on mouse survival and lymphatic contractile function.

## Materials and methods

All animal protocols were approved by the University of Missouri Animal Care and Use Committee and conformed to the National Institutes of Health’s *Guide for the Care and Use of Laboratory Animals* (*8* ^*th*^ *edition, 2011*).

### Mice

*Myh11-CreER^T2^* mice [B6.FVB-Tg (*Myh11*-cre/ERT2)1Soff/J] were originally obtained from Stefan Offermanns (Max-Planck Institute, Bad Neuheim). *Itga8-CreER^T2^* mice were obtained from Joe Miano and Lin Gan (Medical College of Georgia at Augusta University [Warthi et al., 2022)]. Both strains were bred with *Ca*_*v*_ *1.2* ^*f/f*^ mice (*Cacna1c* ^tm3Hfm^/J; JAX #024714) or *Rosa26mT/mG* ^*f/f*^ reporter mice (JAX, #007676), to generate *Myh11-CreER^T2^* *;Ca*_*v*_ *1.2* ^*f/f*^, *Itga8-CreER^T2^* *;Ca*_*v*_ *1.2* ^*f/f*^ *, Myh11-CreER^T2^* *;Rosa26mT/mG* ^*f/f*^, and *Itga8-CreER^T2^* *;Rosa26mT/mG* ^*f/f*^mice. The offspring were injected with tamoxifen (10 mg/ml, 100µl i.p.) for consecutive 5 days and allowed to recover for at least 10 days before testing, as previously described (To et al., 2020). *Ca*_*v*_ *1.2* ^*f/f*^ mice were induced using the same protocol. All genotypes were verified by PCR. Mice (18–30 g) of either sex were studied at 2–6 months of age.

### Vessel isolation, pressure myograph, and data acquisition

Mice were anesthetized by intraperitoneal injection of Ketamine/Xylazine (100/10 mg/kg) and placed in the prone position on a heated tissue dissection/isolation pad. The superficial saphenous vein was exposed by a proximal-to-distal incision in the skin over the calf and the popliteal afferent lymphatic vessels on each side of the vein were identified and isolated as previously described (Castorena-Gonzalez et al., 2018b). Each vessel was then pinned with short segments of 40 µm stainless steel wire onto a Sylgard-coated dissection chamber filled with BSA-containing Krebs buffer at room temperature. Once secured, the surrounding adipose and connective tissues were removed by microdissection. An isolated vessel was then transferred to a 3-ml observation chamber on the stage of a Leica inverted microscope, cannulated and pressurized to 3 cm H_2_O using two glass micropipettes (50–60 µm outside diameter). With the vessel pressurized, the segment was cleared of remaining connective and adipose tissue. Polyethylene tubing was attached to the back of each glass micropipette and connected to a computerized pressure controller (Scallan et al., 2013), with independent control of inflow pressure (Pin) and outflow pressure (Pout). To minimize diameter-tracking artifacts associated with longitudinal bowing at higher intraluminal pressures, input and output pressures were briefly set to 10 cm H_2_O at the beginning of every experiment, and the vessel segment was stretched axially to remove any longitudinal slack.

Following this procedure, each lymphatic vessel was allowed to equilibrate at 37°C with inflow and outflow pressures set to 3 cm H_2_O. Constant exchange of Krebs buffer was maintained using a peristaltic pump at a rate of 0.5 ml/min. After temperature stabilized, the vessel typically began to exhibit spontaneous contractions within 10–15 min that stabilized in a consistent pattern within ∼30 min. Custom LabVIEW programs (National Instruments; Austin, TX) acquired real-time analog data and digital video through an A-D interface (National Instruments) and detected the inner diameter of the vessel (Davis, 2005). Videos of the contractile activity of lymphatic vessels were recorded for further analyses under bright-field illumination at 30 fps using a firewire camera (Basler, Graftek Imaging; Austin, TX).

### Assessment of *ex vivo* contractile function and responses to pressure

The contractile parameters of each vessel were characterized at different levels of intraluminal pressure spanning the physiological range from 0.5 to 10 cm H_2_O (in successive steps: 3, 2, 1, 0.5, 3, 5, 8, and 10 cm H_2_O). Spontaneous contractions were recorded at each pressure over intervals of 2–3 min. Wild type popliteal lymphatics typically exhibited their strongest contractions at pressures between 1-2 cm H_2_O and contraction frequency increased 3 to 5-fold over the pressure range tested. During the pressure response protocol, both the input and output pressures were maintained at equal levels so that there was no imposed pressure gradient for forward flow. At the end of every experiment, all vessels were equilibrated by perfusion with calcium-free Krebs buffer containing 3 mM EGTA for 30 min, and passive diameters were obtained at each level of intraluminal pressure.

### Quantification of spontaneous contractions

Once an experiment was completed, internal diameter traces (from a single representative region of interest) and/or videos of spontaneous contractions were analyzed using custom-written LabVIEW programs to detect end diastolic diameter (EDD), end systolic diameter (ESD), and contraction frequency (FREQ, computed on a contraction-by-contraction basis and averaged over a 2–3 min period). These data were used to calculate commonly reported parameters that characterize the lymphatic contractile function:

$$Amplitude\;\left(AMP\right)=EDD-ESD$$ (1)

$$Normalized\;Amplitude=AMP/D_{\mathrm{MAX}}$$ (2)

$$Ejection\;Fraction\;\left(EF\right)=\left[\frac{{EDD}^{2}-{ESD}^{2}}{{EDD}^{2}}\right]$$ (3)

$$Tone=\left(\frac{D_{\mathrm{MAX}}-EDD}{D_{\mathrm{MAX}}}\right)\times 100$$ (4)

$$Fractional\;Pump\;Flow\;\left(FPF\right)=EF∙FREQ$$ (5)

where D_MAX_ represents the maximum passive diameter (obtained after 30-min equilibration in calcium-free Krebs solution) at a given level of intraluminal pressure. Each of these contractile parameters represents the average of all the recorded contractions during the 2–3 min time period corresponding to each level of intraluminal pressure. In control vessels, contraction amplitudes at pressures between 0.5 and 5 cm H_2_O were typically between 20 and 60 μm for a maximal passive diameter of 60–100 μm. In *Cav1.2*-deficient vessels of comparable size, the changes in inner diameter typically were <5 µm and contraction waves (to the extent they were present) were not entrained, i.e., the diameter fluctuations resembled the irregular vasomotion often observed in lymphatic diastole or in arteries. To exclude these events, a value of 5 µm was used as the threshold for determining a valid spontaneous contraction (for determination of FREQ).

### Pump tests

Active lymphatic contractions are critical for pumping lymph against adverse intraluminal pressure gradients (Scallan et al., 2016). To assess the impact of *Cav1.2* deletion on the pump strength of single lymphangions, a second protocol was conducted in segments containing exactly 2 valves, similar to that described previously (Davis et al., 2012; Lapinski et al., 2017; Castorena-Gonzalez et al., 2018a). With Pin held at 1 cm H_2_O, Pout was elevated ramp-wise from 1 to 12 cm H_2_O at a rate of ∼3 cm H_2_O/min while monitoring the behavior of the outflow valve. The outflow valve normally opened and closed during each contraction cycle, alternating with the inflow valve (Davis et al., 2011), but when Pout exceeded the level of internal pressure generated by a spontaneous contraction, the outflow valve failed to open during lymphatic systole. The Pout value at this point in time (minus Pin) corresponded to the “pump limit” of the lymphangion (Lapinski et al., 2017; Castorena-Gonzalez et al., 2018a). We previously showed that simultaneous measurements of valve position and internal pressure (Psn, made near the output valve using a servo-nulling micropipette), confirm that the pump limit, as assessed by the outflow valve behavior, corresponds to the amplitude of the internal pressure spike at the point in time when Psn fails to exceed the value of Pout. Thus, pump limit can be determined from the outflow valve behavior without directly measuring internal pressure.

A variation of this protocol was used in certain instances in which the outflow valve “locked” open during diastole, a behavior that occurs unpredictably, as described in a previous publication (Bertram et al., 2017). Outflow valve “lock” occurs in some vessels during an increasing Pout ramp when the inflow valve remains closed throughout diastole, dictating that the outflow valve remains open (“locked”) throughout the contraction cycle. This behavior is not directly related to pump strength but rather to the chance (50%) that the inflow valve has a lower *ΔP* (Pout—Pin) for closure than the outflow valve at any particular diastolic pressure (Davis et al., 2011). To circumvent this problem in the subset of vessels exhibiting the behavior, we devised an alternate protocol. When the outflow valve transiently closed during lymphatic diastole, due to the suction effect of vessel wall expansion (Jamalian et al., 2017), Pout was stepped rapidly from 1 to 12 cm H_2_O, forcing the outflow valve to remain closed unless the internal pressure generated during systole was sufficient to overcome the level of Pout. Pout was then slowly lowered, ramp-wise, at ∼3 cm H_2_O/min (or in some cases in step-wise increments of 0.5 cm H_2_O) while spontaneous contractions continued to occur, typically at an elevated rate due to the higher Pout level (Scallan et al., 2012). The valve would then open during lymphatic systole at the point corresponding to the pump limit. We confirmed in test vessels that the pump limit determined by this procedure was very close (within 0.3 cm H_2_O) to that determined by the increasing Pout ramp method.

Valve position, either open or closed, was determined from replay of the recorded protocol videos using a LabVIEW program, as described previously (Davis et al., 2012), while maintaining synchronization of the valve position data with the pressure and diameter data. The pump limit was then determined as described above. Pump tests on each vessel were performed at various levels of Pin (which determines the preload) in duplicate or triplicate and the values at each given level of Pin were averaged to give a single pump limit to be used for statistical analysis.

### RNA isolation and real-time PCR

Total RNA was extracted from dissected and cleaned inguinal axillary lymphatic vessels using the Arcturus PicoPure RNA isolation kit with on-column DNase I treatment (Qiagen, Valencia, CA) according to the manufacturer’s instructions. RNA was eluted with 25 μl nuclease-free water. Purified RNA was transcribed into cDNA using High-Capacity cDNA Reverse Transcription kit (ThermoFisher Scientific, Waltham, MA). Real-time PCR was performed on cDNAs prepared from each sample using 2x PrimeTime Gene Expression Master Mix (IDT, Coralville, IA) with predesigned TaqMan probes: Mm.PT.58.33257376.gs for *β-actin* and Mm.PT.58.12188337 for *Cacna1c* (IDT, Coralville, IA). The *Cacna1c* primer targeted a sequence in the floxed region of *Cav1.2.* Real-time PCR protocols were as follows: preheating at 95°C for 3 min, 45 cycles of two-step cycling of denaturation at 95°C for 15 s and annealing/extension steps of 30 s at 60°C. Data collection was carried out using a Bio-Rad CFX 96 Real-Time Detection System (software version Bio-Rad CFX Manager 3.1; Bio-Rad, Hercules, CA, United States). The relative expression level was calculated according to the published 2^−ΔΔCT^ method (Livak and Schmittgen, 2001).

### Determination of recombination efficiency

For assessment of smooth muscle recombination efficiency, cannulated and pressurized popliteal lymphatic vessels were equilibrated in calcium-free solution for 15 min to prevent movement during live imaging. Image stacks were acquired with a Yokagawa CSU-X Spinning Disc Confocal Microscope on an inverted Olympus IX81 with a Hamamatsu Flash4 camera and ×20 dry objective using Metamorph software. Each live vessel acquisition created a Z-stack of images in 1 micron increments from the lower surface of the vessel to its midpoint for both the membrane GFP (488 nm) and membrane tdTomato wavelengths (561 nm).

To assess recombination efficiency in the smooth muscle layer of popliteal lymphatic vessels, we assessed the percent area of the popliteal vessels covered by GFP^+^ lymphatic muscle cells. Maximum projections were made of the GFP and tdTomato image stacks using Image J. A mask of the GFP^+^ lymphatic muscle cells was created, despeckled, and then was used to assess the percent area of the tdTomato maximum projection encompassed by the mask using the Image J threshold and area measure function.

### Solutions and chemicals

Krebs buffer contained: 146.9 mM NaCl, 4.7 mM KCl, 2 mM CaCl_2_·2H_2_O, 1.2 mM MgSO_4_, 1.2 mM NaH_2_PO_4_·H_2_O, 3 mM NaHCO_3_, 1.5 mM Na-HEPES, and 5 mM D-glucose (pH = 7.4). An identical buffer was prepared with the addition of 0.5% bovine serum albumin (“Krebs-BSA”). During cannulation Krebs-BSA was present both luminally and abluminally; during the experiment the abluminal solution was constantly exchanged with Krebs buffer. Ca^2+^-free Krebs was Krebs with 3 mM EGTA replacing CaCl_2_·2H_2_O and used to determine passive diameter. All chemicals were obtained from Sigma-Aldrich (St. Louis, MO), with the exception of BSA (US Biochemicals; Cleveland, OH), MgSO_4_ and Na-HEPES (ThermoFisher Scientific; Pittsburgh, PA).

### Statistical tests

Statistical analyses were performed using Prism9 (Graphpad Software, San Diego, CA). The number *n* refers to the number of vessels included per group. The normality of each data set was first tested to determine whether parametric or non-parametric tests should be used. Statistical differences in the various contraction parameters were tested at each pressure level between male and female vessels and between *Cav1.2* ^*f/f*^ and *Itga8-CreER^T2^* *;Cav1.2* ^*f/f*^ vessels using two-way ANOVAs with repeated measures and Tukey’s multiple comparison tests. A one-way ANOVA with Kruskal-Wallis non-parametric tests and Dunn’s multiple comparison tests were used for the analysis of pump tests, as 3 of the 4 tests for normality in Prism indicated that the pump test data were not normally distributed. Data are plotted as mean ± SEM with significance set at *p* < 0.05 unless otherwise stated.

## Results

### Survival analysis

**FIGURE 1:**
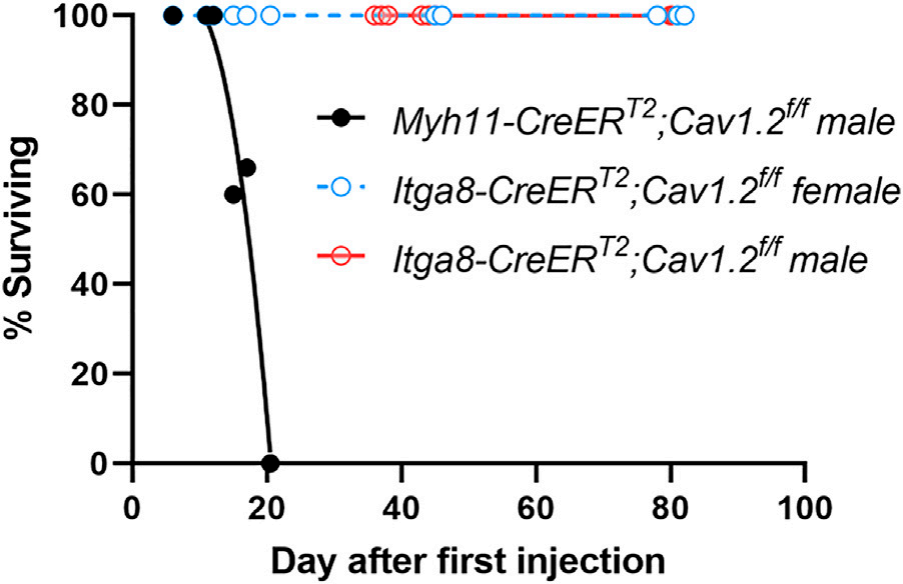
Survival curves for 21 *Myh11-CreER^T2^;Cav1.2^f/f^* mice and 21 *Itga8-CreER^T2^;Cav1.2^f/f^* mice, with the latter separated by sex. Curve fits were made using non-linear regression.

We compared the survival of *Itga8-CreER^T2^*;*Cav1.2^f/f^* mice with *Myh11-CreER^T2^* *;Cav1.2* ^*f/f*^ mice after the start of the tamoxifen injection protocol (Figure 1). *Myh11-CreER^T2^* *;Cav1.2* ^*f/f*^ mice began to die ∼day 15 after the first injection and no *Myh11-CreER^T2^* *;Cav1.2* ^*f/f*^ mice survived past day 20 post-induction, compared to 100% (5/5) male *Itga8-CreER^T2^* *;Cav1.2* ^*f/f*^ mice that were still alive at the time of sacrifice on day 78–82 post-induction and (1/1) female *Itga8-CreER^T2^* *;Cav1.2* ^*f/f*^ mice that was alive at the time of sacrifice on day 80 post-induction. Additionally, all *Itga8-CreER^T2^* *;Cav1.2* ^*f/f*^ mice were in good health and continued to feed and gain weight normally up until the day of sacrifice.

### Analysis of Cre efficiency

**FIGURE 2:**
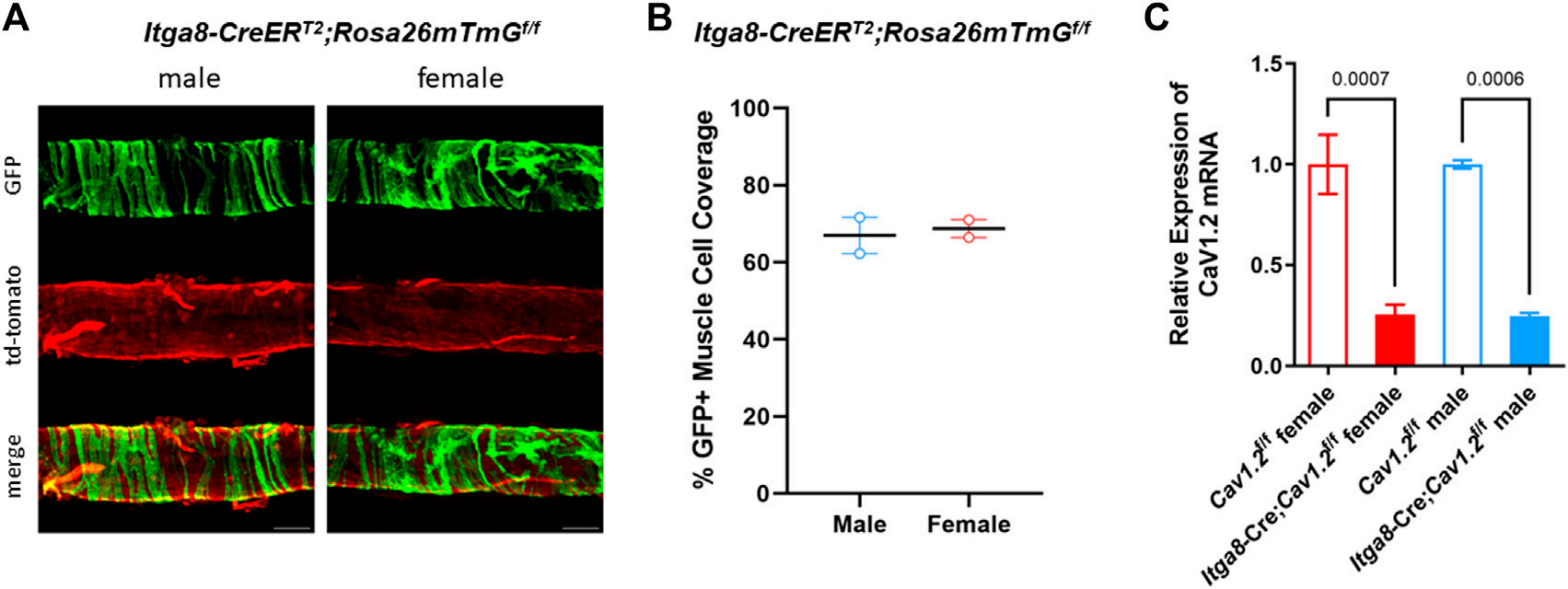
Evaluation of recombination efficiency. **(A)** Images of GFP and td-tomato expression in male and female inguinal-axillary lymphatics from *Itga8-CreER^T2^*;*Rosa26mTmG^f/f^* mice, showing recombination (GFP + cells) in the majority of lymphatic smooth muscle cells. **(B)** Summary of %GFP + cells in segments of 2 male and 2 female inguinal-axillary lymphatics from *Itga8-CreER^T2^*; *Rosa26mTmG^f/f^* mice. **(C)** Expression of *Cav1.2* mRNA relative to β-actin from inguinal-axillary vessels of 3 male and 3 female *Cav1.2^f/f^* mice and 3 male and 3 female *Itga8-CreER^T2^;Cav1.2^f/f^* mice.

We recently reported the efficiency of recombination for the *Rosa26 mT/mG* reporter in lymphatics from *Myh11-CreER^T2^* *;Rosa26mT/mG* ^*f/f*^ vs. *Itga8-CreER^T2^* *;Rosa26mT/mG* ^*f/f*^ mice [Figure 3 in ref (Warthi et al., 2022)]. Here, we reanalyzed a subset of that data (2 male *Itga8-CreER^T2^* *;Rosa26mT/mG* ^*f/f*^ mice) along with data from two (new) female *Itga8-CreER^T2^* *;Rosa26mT/mG* ^*f/f*^ mice to compare the recombination efficiency in lymphatics from mice of different sexes. Fluorescence images of GFP in pressurized popliteal lymphatics are shown in Figure 2A. GFP expression is evident in the circumferential (i.e., smooth muscle) layer of cells in both male and female vessels. Quantification of recombination efficiency was similar between male and female vessels, as shown in Figure 2B. Because it is well known that recombination efficiency depends not only on the particular Cre used, but also on the length of the floxed gene (Stifter and Greter, 2020), we assessed the degree of *Cav1.2* knock down by *Itga8-CreER^T2^* in male and female inguinal-axillary lymphatics using qPCR. As quantified in Figure 2C, *Cav1.2* levels were decreased by ∼75% from control in both male and female lymphatic vessels. The actual degree of knock down of *Cav1.2* may have been even greater because the qPCR results represent message from all cell types in the vessel wall that may also express *Cav1.2*, including non-SM cells such as neurons and some immune cells.

### Analysis of spontaneous contractions

The absence of propulsive, spontaneous contractions in popliteal lymphatic vessels from *Myh11-CreER^T2^* *;Cav1.2* ^*f/f*^ mice was described in a previous publication (To et al., 2018). In that study, normal contractions, with amplitudes of 40–60 μm at pressures 0.5–3 cm H_2_O, were recorded in 7 of 7 popliteal vessels from *Cav1.2* ^*f/f*^ mice but were absent in 5 of 5 vessels from *Myh11-CreER^T2^* *;Cav1.2* ^*f/f*^ mice [see Figure 12 in (To et al., 2018)]. The residual contractile activity observed in *Myh11-CreER^T2^* *;Cav1.2* ^*f/f*^ vessels consisted only of small (<5 μm amplitude), non-entrained diameter oscillations with an irregular frequency, and neither the frequency nor amplitude were highly regulated by pressure as in WT or *Cav1.2* ^*f/f*^ vessels (To et al., 2018). However, a subsequent analysis of popliteal lymphatic contractions in another cohort of *Myh11-CreER^T2^;Cav1.2^f/f^* mice, studied at the same time point after tamoxifen induction, revealed that 2 of 7 vessels did exhibit small (5–12 μm) contractions at certain low pressures (Zawieja et al., 2022).

**FIGURE 3:**
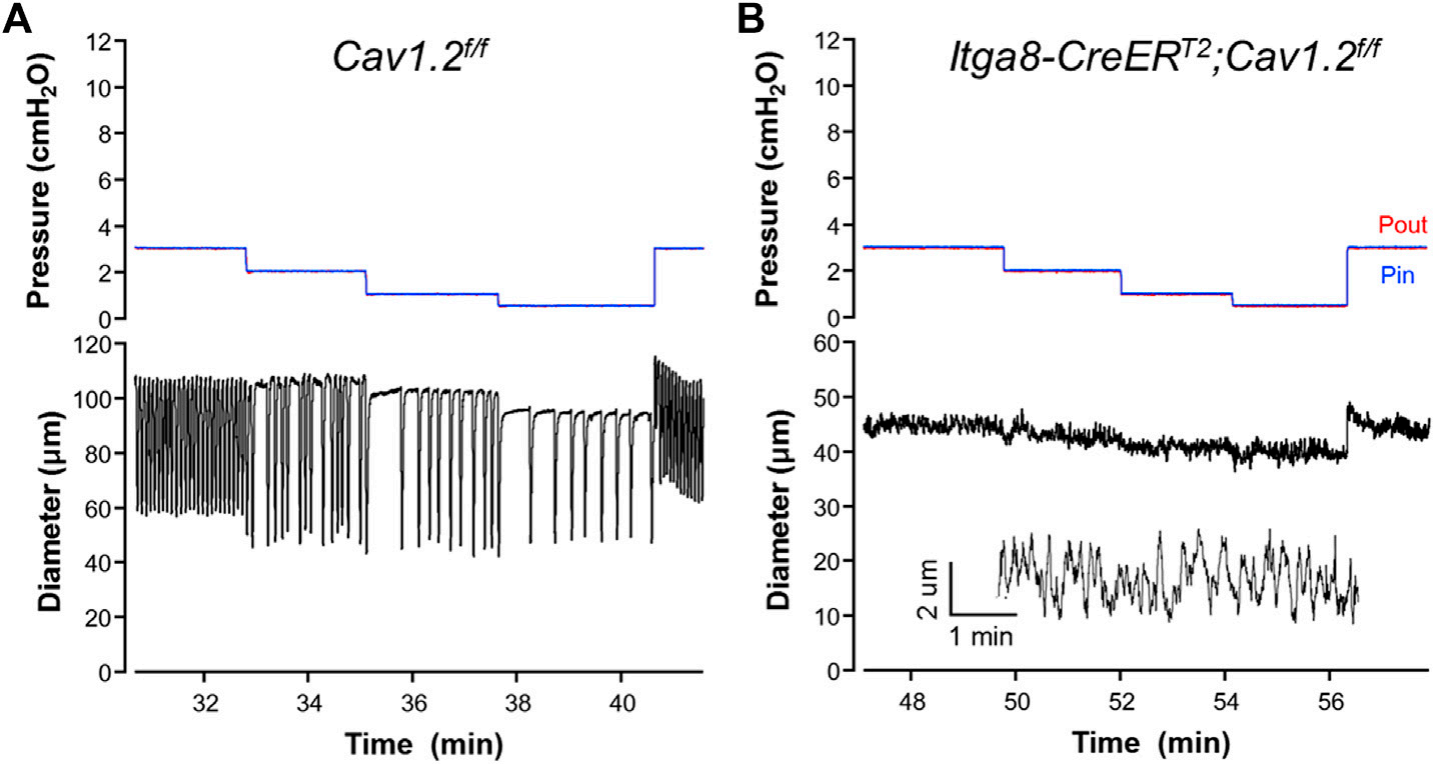
Representative recordings of contractile activity of popliteal lymphatic vessels from **(A)** a *Cav1.2^f/f^* mouse and **(B)** a *Itga8CreER^T2^;Cav1.2^f/f^* mouse. Pin and Pout were kept at equivalent levels throughout the protocols to avoid any net changes in flow. The inset in **(B)** is an expanded section of the diameter trace at pressure = 3 cmH_2_O.

We analyzed the contractile activity of popliteal lymphatic vessels from *Itga8-CreER^T2^* *;Cav1.2* ^*f/f*^ mice and a new cohort of *Cav1.2* ^*f/f*^ mice after tamoxifen induction. Representative examples of spontaneous contractions observed in vessels from the two strains of mice are shown in Figure 3. Robust contractions were observed at all pressures between 0.5 and 10 cm H_2_O in popliteal vessels from *Cav1.2* ^*f/f*^ mice, similar to results we have reported in multiple studies from other tamoxifen-treated control vessels (Castorena-Gonzalez et al., 2018a; To et al., 2018; Zawieja et al., 2018). The contraction amplitude in the *Cav1.2* ^*f/f*^ vessel shown in Figure 3A varied between 44 and 64 μm depending on the pressure level, with a maximum amplitude at 2 cm H_2_O. As before, contraction frequency was highly sensitive to pressure such that frequency decreased from 12 min^−1^ at 3 cm H_2_O to 3 min^−1^ at 0.5 cm H_2_O. In this vessel, a maximal frequency of 14 min^−1^ was reached at 5 cm H_2_O; thus, frequency varied 4.6-fold over this pressure range. In contrast, the popliteal lymphatic from an *Itga8-CreER^T2^* *;Cav1.2* ^*f/f*^ mouse only exhibited contractions less than 3 μm in amplitude at all pressures (Figure 3B), with an estimated diameter oscillation frequency of ∼10 min^−1^ at all pressures (see insert showing zoomed diameter trace).

**FIGURE 4:**
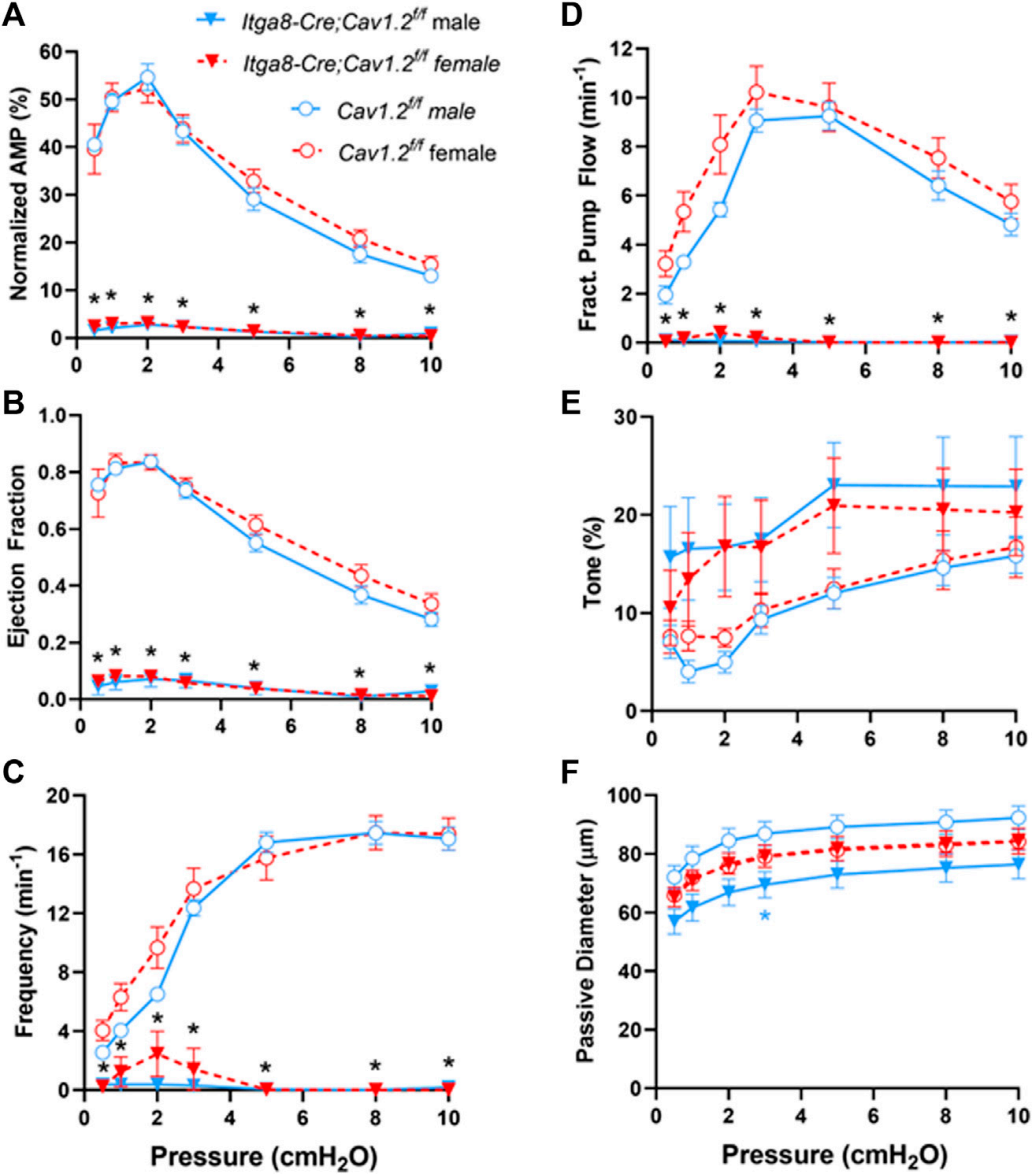
Summary of contractile parameters, **(A)** Normalized Amp, **(B)** EF, **(C)** Frequency, **(D)** FPF, **(E)** Tone, **(F)** Passive Diameter, for popliteal lymphatic vessels from *Cav1.2* ^*f/f*^ mice and *Itga8-CreER^T2^* *;Cav1.2* ^*f/f*^ mice at various pressures from 0.5 to 10 cmH_2_O. The parameters and statistical tests were determined as described in Methods. Black asterisks indicate significant differences in parameters both between 10 male *Cav1.2* ^*f/f*^ and 8 male *Itga8-CreER^T2^* *;Cav1.2* ^*f/f*^ vessels and between 10 female *Cav1.2* ^*f/f*^ and 9 female *Itga8-CreER^T2^* *;Cav1.2* ^*f/f*^ vessels (*p* < 0.05). Blue asterisks indicate significant differences between only male *Cav1.2* ^*f/f*^ and male *Itga8-CreER^T2^* *;Cav1.2* ^*f/f*^ parameters (*p* < 0.05).

The average contraction parameters from popliteal lymphatic vessels from 4 male and 5 female *Cav1.2* ^*f/f*^ mice and from 4 male and 4 female *Itga8-CreER^T2^* *;Cav1.2* ^*f/f*^ mice are summarized in Figure 4. Vessels from both male and female mice were evaluated separately. Two-way ANOVAs with repeated measures were used to assess possible differences in contractile parameters (normalized amplitude, frequency, ejection fraction, tone, FPF and passive diameter) of popliteal lymphatics from *Cav1.2^f/f^* mice. No significant differences were found between the parameters of male and female vessels. Likewise, two-way ANOVAs with repeated measures were used to assess possible differences in contractile parameters of popliteal lymphatics from *Itga8-CreER^T2^* *;Cav1.2* ^*f/f*^ mice. No significant differences were found between the parameters of male and female vessels. However, there were significant impairments in all contraction parameters, except tone, at every pressure in vessels from *Itga8-CreER^T2^* *;Cav1.2* ^*f/f*^ mice compared to *Cav1.2^f/f^* mice. The normalized contraction amplitudes of *Cav1.2* ^*f/f*^ control vessels were consistent with those reported in our previous studies (Castorena-Gonzalez et al., 2018a; To et al., 2018; Zawieja et al., 2018), with a peak AMP at pressures of 1-2 cm H_2_O, a slight reduction at 0.5 cm H_2_O and progressive decline at pressures >2 cmH_2_O (Figure 4A). In contrast, normalized contraction amplitudes of *Cav1.2*-deficient vessels were almost negligible at all pressures. The calculated EF of control vessels exceeded 0.8 at low pressures and declined to 0.35 as pressure was elevated to 10 cm H_2_O. EF of *Cav1.2-*deficient vessels was <0.1 at all pressures (Figure 4B). The contraction frequency of control vessels increased from ∼3 min^−1^ to ∼16 min^−1^ over the pressure range 0.5–10 cm H_2_O, in contrast to *Cav1.2*-deficient vessels, in which threshold contractions were rare (Figure 4C). 5 of 8 male vessels and 6 of 9 female vessels from *Itga8-CreER^T2^* *;Cav1.2* ^*f/f*^ mice had no contractions larger than 4 μm at any pressure such that the average FREQ was <2 min^−1^ at all pressures. Calculations of FPF, from EF and FREQ, predicted that *Cav1.2*-deficient vessels were incapable of actively transporting lymph over the entire pressure range (Figure 4D). Interestingly, the tone of *Cav1.2*-deficient lymphatic vessels was 50–75% higher than that of control vessels at most pressures, although none of differences were statistically significant (Figure 4E). The same pattern was observed previously in *Myh11-CreER^T2^* *;Cav1.2* ^*f/f*^ vessels (To et al., 2018), although tone in that study was even higher in *Cav1.2-*deficient vessels (reaching ∼30%) and was significantly different at most pressures. The passive diameters of *Itga8-CreER^T2^* *;Cav1.2* ^*f/f*^ popliteal lymphatics were not significantly different than those of control popliteal lymphatics, with a single exception for male mice at one pressure (Figure 4F).

### Pump limit analysis

**FIGURE 5:**
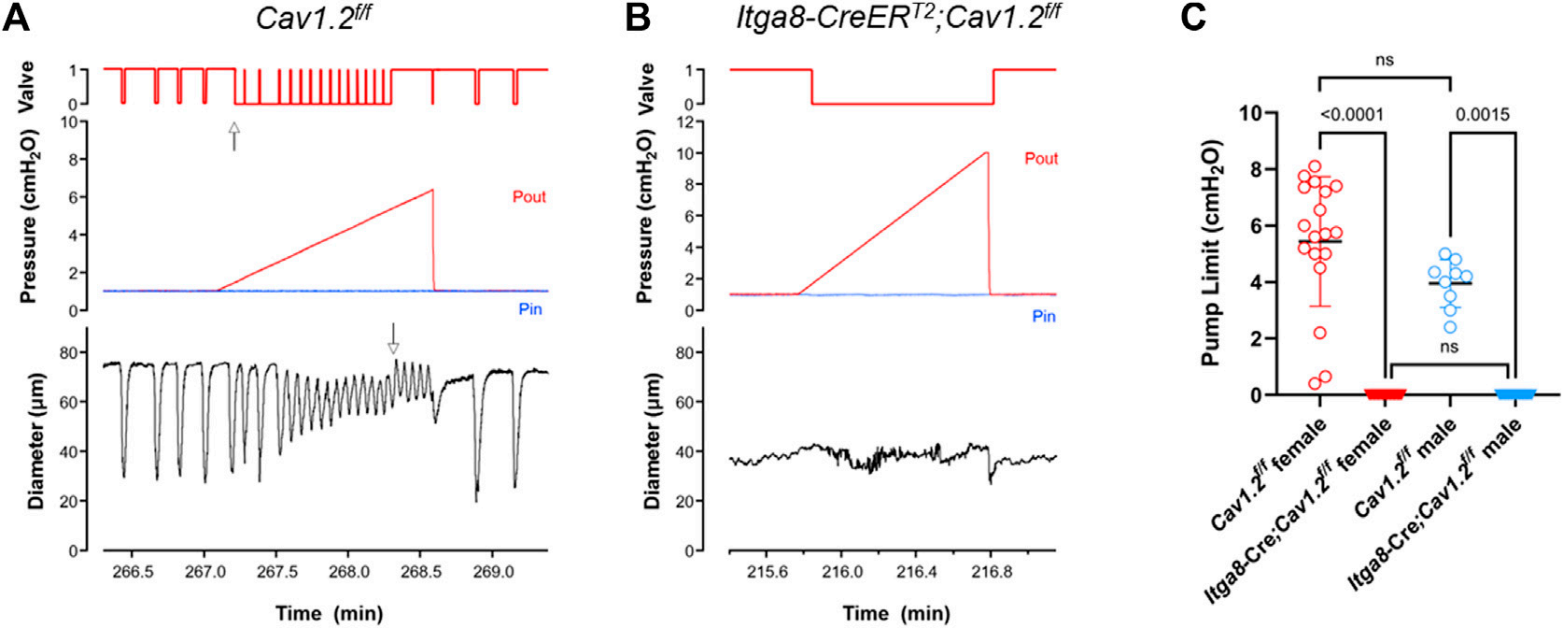
Pump tests for 2-valve popliteal lymphatic segments from *Cav1.2* ^*f/f*^ mice and *Itga8-CreER^T2^* *;Cav1.2* ^*f/f*^ mice. **(A)** Representative pump test for a *Cav1.2* ^*f/f*^ popliteal vessel, showing the output valve behavior (opening, closing with each contraction cycle even as Pout was elevated ramp-wise). **(B)** The output valve in a *Itga8-CreER^T2^* *;Cav1.2* ^*f/f*^ popliteal vessel never closed when pressures were equal and never opened when Pout > Pin. Valve position: 1 = open; 0 = closed. Up arrow in **(A)** indicates point in time when valve is predominantly closed during the contraction cycle (during the Pout ramp) and opens only at the peak of lymphatic systole. Down arrow in **(A)** indicates the point in time when the output valve locked open, immediately after the last ejection during lymphatic systole; the pump limit at that instant, as determined from Pout, was 5.3 cmH_2_O. Pout = red lines; Pin = blue lines. **(C)** Summary of pump test values from 3 male and 8 female *Cav1.2* ^*f/f*^ vessels and 4 male and 6 female *Itga8-CreER^T2^* *;Cav1.2* ^*f/f*^ vessels.

Examples of pump tests in control and *Cav1.2*-deficient vessels are shown in Figures 5A, B. In the control vessel, the valve trace indicates that the outflow valve was open (position = 1) most of the time during the lymphatic contraction cycle (prior to the start of the Pout ramp). This agrees with our previous finding that the valves have an open bias in the absence of a trans-valve pressure difference. The valve closed (position = 0) transiently near the end of diastole in each contraction cycle, which we previously showed corresponds with a transient dip in internal pressure (the “suction effect”) (Jamalian et al., 2017). Shortly after the start of the Pout ramp, when Pout exceeded Pin by ∼0.3 cm H_2_O, the valve switched to the closed state for the majority of the contraction cycle (up arrow, Figure 5A), except for a brief period at the peak of systolic ejection, when it transiently opened. This pattern continued to occur as Pout progressively increased, until a point was reached when the valve ‘locked’ open in diastole (down arrow, Figure 5A). The value of Pout associated with the last successful ejection (minus Pin) was an estimate of the pump limit (and probably an underestimate of the true value due to valve “lock”). In contrast, the outflow valve was open initially in a 2-valve popliteal vessel from an *Itga8-CreER^T2^* *;Cav1.2* ^*f/f*^ mouse, never closed transiently during the contraction cycle at equal Pin and Pout, closed when Pout exceeded Pin, and never opened until the Pout ramp was terminated (Figure 5B). This behavior is indicative of a completely ineffective active pump.

Pump limit values for 2-valve lymphangions from 3 male and 3 female *Cav1.2* ^*f/f*^ mice and 4 female and 2 male *Igta8-CreER^T2^* *;Cav1.2* ^*f/f*^ mice are summarized in Figure 5C. The data from male and female mice were tested using a one-way ANOVA using a Kruskal-Wallis nonparametric test and Dunn’s *post hoc* tests. There were no significant differences between male and female vessels for either of the two genotypes, but the differences between control and Cav1.2-deficient vessels were highly significant for both male and female mice. None of the vessels from *Igta8-CreER^T2^* *;Cav1.2* ^*f/f*^ mice were able to generate sufficient pump strength to open an output valve at any value of Pout, confirming that active lymphatic pumping is eliminated by *Cav1.2* deficiency.

## Discussion

Our results suggest that *Itga8-CreER^T2^* is as effective as *Myh11-CreER^T2^* in deleting *Cav1.2* from lymphatic smooth muscle, with comparable efficiency in male and female mice. Contraction parameters for popliteal lymphatic vessels from *Itga8-CreER^T2^* *;Cav1.2* ^*f/f*^ mice in the present study were comparable to those in popliteal vessels from *Myh11-CreER^T2^* *;Cav1.2* ^*f/f*^ mice in a previous study (To et al., 2018) in that propulsive contractions were completely absent. Most spontaneous contractile activity in lymphatic vessels of both strains of *Cav1.2*-deficient mice consisted of un-entrained diameter oscillations with amplitudes <5 μm. The few residual contractions were infrequent, much smaller than normal, and apparently ineffective at propelling lymph. While pump tests were not performed in our previous study of *Myh11-CreER^T2^* *;Cav1.2* ^*f/f*^ mice, calculations of FPF in vessels from that *Cav1.2*-deficient strain were comparable to those shown here, and FPF was sufficiently low to predict there would be no active lymph propulsion. Pump tests in the present study revealed that none of the *Itga8-CreER^T2^* *;Cav1.2* ^*f/f*^ vessels tested could generate a propulsive contraction against even a slightly elevated Pout level. Taken together with the expression data in Figure 2, these observations also suggest that submaximal recombination of the *Rosa26mTmG* reporter driven by either of the two different Cre lines tested does not preclude effective deletion of *Cav1.2* in lymphatic SM. A similar conclusion regarding deletion of the *Srf* gene was reached in the study by Miano and colleagues (Warthi et al., 2022), and locus-specific or flox-site disparities in recombination efficiency with inducible Cre lines has been widely reported (Stifter and Greter, 2020).

Critically, as with *Myh11-CreER^T2^*;*Cav1.2^f/f^* mice, *Itga8-CreER^T2^* *;Cav1.2* ^*f/f*^ mice did not exhibit any signs of peripheral edema (e.g., footpad swelling) even in the complete absence of active lymphatic contractions. Possible explanations are that a gravitational load in the mouse is required in order for lymphedema to develop or that the presence of functional secondary valves combined with elevated vessel tone is sufficient to prevent lymphedema. We also previously failed to detect any evidence for hindlimb edema in connexin45-deficient mice, even with the mice oriented for short periods of time in a vertical body position (Castorena-Gonzalez et al., 2018a), and this finding is consistent with similar observations in *Foxc2*- or *Rasa1-*deficient mice (Lapinski et al., 2012; Burrows et al., 2013; Sabine et al., 2015), even though the homologous mutations in humans cause primary lymphedema (Eerola et al., 2003; Mellor et al., 2007; Ferrell et al., 2010; Mellor et al., 2011; Ostergaard et al., 2011; Brice et al., 2013; Revencu et al., 2013). Thus, it is possible that a *chronic* gravitational load would be required to overwhelm a mouse lymphatic system with impaired contractile function.

A primary advantage of *Itga8-CreER^T2^* over *Myh11-CreER^T2^* for lymphatic studies is the potential lack of complications from gastrointestinal SM myopathy when certain genes are deleted. Deletion of critical genes such as *Srf* or *Cav1.2* from visceral SM leads to a rapid decline in the health of the animals that could potentially confound the interpretation of lymphatic and vascular SM studies. The other advantage of using *Itga8-CreER^T2^* mice is that it allows comparisons of male-female differences. We tested both male and female mice in the present study, and statistical analyses indicated that *Itga8-CreER^T2^* produced equivalent recombination in both male and female mice and that there were comparable losses of contractile activity and pumping ability of *Cav1.2*-deficient lymphatic vessels from both sexes.

In conclusion, *Itga8-CreER^T2^* provides an effective method to delete genes in lymphatic SM, avoiding potentially lethal visceral myopathy and allowing comparative studies of lymphatic contractile function in both male and female mice. Ideally, a Cre line could be developed that is specific for lymphatic SM, thus avoiding effects on the systemic blood vasculature. However, this would likely require comparative studies of genes expressed differentially in vascular, lymphatic and visceral SM in order to identify a lymphatic SM-specific promoter. To our knowledge, such studies have not yet been performed.

## Data Availability

The raw data supporting the conclusion of this article will be made available by the authors, without undue reservation.
